# Operations for the Cure of Chronic Empyema of the Antrum

**Published:** 1899-03

**Authors:** H. J. Custer

**Affiliations:** Columbus, O.


### THE DENTAL REGISTER.

VOL. LIII.] MARCH, 1899. [No. 3.

### COMMUNICATIONS.

### Operations for the Cure of Chronic Empyema of the Antrum.

H. J. CUSTER, B.S., D.D.S., COLUMBUS, O.

Read at Ohio State Dental Society, December, 1898.

Explanatory of my topic, it would appear well to say that the first and most important part of my paper consists of the description of an operation for the rapid cure of chronic empyema of the antrum, as performed by Dr. Luc, of Paris.

Following a discussion of Dr. Luc's method, I shall give the technic of an operation, the component details of which I have performed in different cases, but have not yet combined in any one case.

Dr. Luc's operation differs widely from others and was first performed by him about two years ago, but I believe, however, that practically the same operation was introduced some four years ago, by a New York surgeon whose identity I am unable to establish.

The chief advantages of the method are, the thorough inspection afforded of the antrum, and the removal of fungosities and other obstacles usually found in chronic empyema, while no obnoxious materials can pass from mouth into antrum, or medicaments in the opposite direction, as occurs in some other methods.

Dr. Luc operates by making a horizontal incision, 1½ centimeters long, in the canine fossa, on a level with the antral floor. From each end of this incision another extends upwards about the same distance. The mucous membrane, with periosteum, is now detached from the bone, which with the antral membrane is removed by a bur, chisel and mallet, or cutting forceps. Once inside the antrum, the fungosities are removed by means of a sharp curette. This curetting usually causes rather brisk hemorrhage, which is controlled by tamponing with gauze.

After curetting, the antrum is tamponed for a few minutes with 20 percent chlorid of zinc gauze, to destroy any particles of vegetations that may have escaped the curette.

In order to provide for drainage and irrigation, an opening, one centimeter in diameter, is made through the internal wall of the antrum, at its most anterior point, that is on a level with the floor of the nasal fossa. This opening is made by working through the antrum, and requires the cheek to be strongly retracted.

Dr. Luc originally operated by opening the cheek from the commissure back to the masseter muscle, but he now operates without external incision.

The drainage tube passes through this opening into the inferior meatus, with the end appearing at the nostril. The tube used is soft gum, ¼ inch in diameter, with a funnel-shaped expansion on one end. This expanded end, placed within the antrum, serves to keep the tube in position and yet allow easy removal when a cure is affected.

When the tube is in position the buccal wound is closed by suturing the mucous membrane with five or six fine gut sutures. Quietude and liquid diet are maintained for about three days, when the mucous membrane is found united and irrigations are begun.

Daily irrigations of either a saturated solution of boracic acid, or a 1-2000 solution of formol, followed by c. c. of iodoform in ether 1.10, injected through the drain by means of a long-nozzled syringe, constitute the treatment while the tube is in position.

After from two to four weeks the drain is removed and the same agents, only with increasing interval, are continued through the nasal opening, until discharge ceases. On an average a cure is effected in six weeks.

This is not a very rapid cure in comparison with those cases reported cured in ten to fourteen days by irrigating through a small perforation, or even by the natural opening. Those rapid cures by simpler means in all probability occur in those cases whose pyogenic membrane or fungosities are not pronounced, and where the antrum is free from septa, polypi, and other obstacles. In this connection, it is well to remark that Fillebrown has shown how a discharge from a frontal sinus is liable to find its way into the antrum. This fact should not be forgotten in treating any obstinate case of pyemia.

My notes do not give a percentage on Luc's entire clinic, but I was informed that fungosities in chronic empyema were the rule. Among nine antres that were opened, on various occasions, I have seen marked fungosities in six cases, in two of which, the antres were nearly filled. One antrum with and one without fungosities had bony septa that required removal, and one case with fungosities had also mucous polypi. Altogether I have been identified with sixteen cases, including four in my own practice, most of the remaining having been in the foreign clinics. These cases were treated either by the natural opening, by artificial opening into the nasal fossa, by small and temporary opening through alveolar process or canine fossa, or by large permanent opening in canine fossa. Among this number only one cure was affected within a month.

Without doubt some cases of chronic empyema are cured in two to four weeks by proper opening through the mouth or nasal fossa, or even by the natural opening, without inspection of the antrum. But not infrequently the treatment extends over several months and sometimes the case will not yield at all without surgical removal of various obstacles that occur within the antrum.

Those of us who have treated an alveolar abscess with a series of injections, without success, and then have attained a rapid cure after the surgical use of a sharp bur, can readily appreciate the value of removing abnormal conditions within the antrum by surgical means.

Considering also the good results following the judicious use of the curette in the mastoid and frontal sinuses, as well as in general surgery, I should feel justified in making free temporary opening, other things equal, in any case in which the circumstances of the patient required speedy cure; in any case in which the use of ordinary measures prove ineffectual; or in case diagnosis was uncertain, and positive diagnosis does not always obtain, as is forcibly illustrated in those cases related by Heath, in which some eminent surgeons suspecting malignant growth, have begun the removal of the jaw, when the trouble was chronic empyema.

By inspection of the antrum we learn the most favorable place for drainage; we learn the condition of the ostium and perhaps find a foreign body, fungosities, bony septa, either partial or complete, mucous polypi, dentigerous and other cysts, necrosed bone, or benign or malignant tumors.

Some of these would certainly and others probably require removal for the welfare of the patient.

Regarding the drainage system of Dr. Luc, whom I greatly respect, both as a gentleman and as a surgeon, it seems, for reasons appearing later, that drainage through the inferior meatus is not so competent as a proper drain through the mouth.

I have searched to some extent for statistcs regarding the relative position of antral and nasal floors, but without success. However, I have examined a number of skulls, which with six observations made during life, make a total of 33 antres inspected. Among this number four showed the antral and nasal floors to be about even, while among the remaining 29 the floor of the antrum was lower than that of the nasal fossa. The depression of the antral floor was most marked in cases with high palate. The difference between the two floors, in one instance, amounted to about half an inch. Furthermore, among all illustrations that I could find, no illustration was observed showing the antral floor above the nasal. Therefore we are certain that in some cases, at least, drainage through the inferior meatus is not the best route so far as drainage downward is concerned, while the tube in the nose, is, I think, more inconvenient than a proper metal one in the mouth.

Regarding the flap that is lifted in opening the antrum, the question may be asked as to whether the entire thickness of the antral wall could not be successfully turned upward instead of cutting away the bone. In the case of a healthful young man whose first molar palatine tooth had been forced into the antrum, the previous day, during attempted extraction, I cut through the entire wall in the canine fossa, with a thick scalpel, carefully guarded. I then turned the rectangular flap upwards until the bone fractured. The root was then secured, the antrum washed out with a warm solution of Listerine, the flap pushed back into position and the mucous membrane satured with silk. As the flap bulged outward at the bottom, an iodoform pad was placed over it with pressure added by a firm bandage around the face. Four days later the flap was firm and more than a year later all appeared to be satisfactory.

Whether the bone can be saved when the lining of the antrum is damaged, as in chronic empyema, along with the attending debility, I am not prepared to say.

At present I prefer to proceed with the following method: First, sterilize the mouth by brushing with Listerine, because it is a good solvent and carries obnoxious materials away bodily. They use corrosive sublimate, 1-4000, to destroy remaining bacteria, and push the ether to prevent vomiting. Draw the cheek well out of the way by means of a retractor placed in the commissure, and not held by an assistant, but buckled to a strong cap tightly fitted to the head.

Begin a quarter of an inch above the first bicuspid root and cut through mucous membrane and periosteum, straight backwards for 1½ centimeters; then upwards 1 centimeter from each extremity. Lift the periosteum and perforating the bone with a drill at the four anlges, remove the denuded bone along with antral membrane, with a saw or thin-beaked cutting forceps. Use a moderately sharp curette to remove fungosities or polypi with small pedicles, and if there be any bony septa that require removal, use the dental bur, carefully avoiding any conical prominences over roots of teeth. Tampon thoroughly with 20 percent zinc chlorid gauze, to destroy remnants of pyogenic membrane, and to stop hemorrhage in the meantime. During the interval establish drainage by drilling through the alveolar process, if that be the proper position, and fit the tube into place.

Remove the gauze, and if bleeding recurs, tampon tightly again for a few minutes with gauze wrung out of hot water. Then close antrum by stitching mucous membrane with five or six fine gut sutures.

Maintain quietude, and liquid diet through a tube, until about the third day when the flap should be united and irrigations. For the first two weeks I prefer a saturated solution of boracic acid—twice daily, followed by 2 c. c. of iodoform in ether 1-10 once daily. Later, increase the interval.

As to the exact place for drainage, that is determined principally by inspection of the antrum. If the dental arch be intact, or if a tooth remain to which the tube can be attached, I would then make a groove in the original opening, on a level with the antral floor, and cementing the lower end of the tube to a tooth, by means of a cap or band, stitch the flap around the end entering the antrum. Or, if the most dependent part of the antrum was over the place of a tooth that had been or should be extracted, I would then place the tube through the entire thickness of the alveolar process. If the patient wore a plate, I would still prefer the alveolar ridge, because it will accommodate a tube of large capacity. I would then make a depression in the plate to receive the end of the tube, and direct the patient to wear the plate during sleep. If the jaw were edentulous and no plate worn, the dental ridge would still be my choice, and I would have a plate made to wear over the tube.

The drainage tube is one of my own device, that I began using about six years ago. For the alveolar process it is a round tube extending into the antrum $\frac{1}{8}$ inch, to prevent occlusion by swelling of lining membrane. This last $\frac{1}{8}$ inch is perforated to allow drainage into the tube, which is closed at the lower end, but not by a solid plug. For this purpose I use an inner tube closed at one end, similar to a cartridge shell. In this appliance I have a metallic reservoir, into which the discharge runs and from which it can be removed by the patient several times a day, and that without contaminating the mouth.

Originally I used this tube $\frac{1}{8}$ inch in diameter, but now I prefer $\frac{1}{4}$ inch diameter, to secure greater capacity.

When drainage is through the canine fossa the antral end bends inward and is $\frac{1}{8}$ inch in diameter, with a broad flat expansion, which lies over the alveolar process, to serve as a reservoir. This, also, is closed by an inner tube, and is within easy reach of the patient.

The advantages claimed for the operation after my modification, are, that perfect drainage is secured while the antrum is closed against infection from without; the irrigation and discharge can be placed under control of the patient; in some instances a special opening for a drain is not required; and finally, the operation is all through the mouth, by which route it can be performed by the dentist, who familiar with economy of space would seldom think of external incision with the resulting scar, as I have repeatedly seen in oral surgery practiced by the general surgeon.

